# Promising Efficacy of a Third Dose of mRNA SARS-CoV-2 Vaccination in Patients Treated with Anti-CD20 Antibody Who Failed 2-Dose Vaccination

**DOI:** 10.3390/vaccines10060965

**Published:** 2022-06-17

**Authors:** Yohei Funakoshi, Kimikazu Yakushijin, Goh Ohji, Wataru Hojo, Hironori Sakai, Marika Watanabe, Akihito Kitao, Yoshiharu Miyata, Yasuyuki Saito, Shinichiro Kawamoto, Katsuya Yamamoto, Mitsuhiro Ito, Taiji Koyama, Yoshinori Imamura, Naomi Kiyota, Hiroshi Matsuoka, Yasuko Mori, Hironobu Minami

**Affiliations:** 1 Division of Medical Oncology/Hematology, Department of Medicine, Kobe University Hospital and Graduate School of Medicine, Kobe 650-0017, Japan; kyakushi@med.kobe-u.ac.jp (K.Y.); mwat624@med.kobe-u.ac.jp (M.W.); akitao@med.kobe-u.ac.jp (A.K.); kyamamo@med.kobe-u.ac.jp (K.Y.); hameyama@med.kobe-u.ac.jp (T.K.); yimamura@med.kobe-u.ac.jp (Y.I.); nkiyota@med.kobe-u.ac.jp (N.K.); hminami@med.kobe-u.ac.jp (H.M.); 2 Division of Infection Disease Therapeutics, Department of Microbiology and Infectious Diseases, Kobe University Hospital and Graduate School of Medicine, Kobe 650-0017, Japan; ohji@med.kobe-u.ac.jp; 3 R&D, Cellspect Co., Ltd., Morioka 020-0857, Japan; whoujou@cellspect.com (W.H.); hsakai@cellspect.com (H.S.); 4 BioResource Center, Kobe University Hospital, Kobe 650-0047, Japan; yhmiyata@med.kobe-u.ac.jp (Y.M.); matsuoh@med.kobe-u.ac.jp (H.M.); 5 Division of Molecular and Cellular Signaling, Kobe University Graduate School of Medicine, Kobe 650-0017, Japan; ysaito@med.kobe-u.ac.jp; 6 Department of Transfusion Medicine and Cell Therapy, Kobe University Hospital, Kobe 650-0017, Japan; skawamo@med.kobe-u.ac.jp; 7 Division of Medical Biophysics, Kobe University Graduate School of Health Sciences, Kobe 654-0142, Japan; itomi@med.kobe-u.ac.jp; 8 Cancer Center, Kobe University Hospital, Kobe 650-0017, Japan; 9 Division of Clinical Virology, Center for Infectious Diseases, Kobe University Graduate School of Medicine, Kobe 650-0017, Japan; ymori@med.kobe-u.ac.jp

**Keywords:** SARS-CoV-2 vaccination, booster shot, anti-CD20 antibody, B-cell malignancies

## Abstract

Anti-CD20 antibodies react with CD20 expressed not only on malignant B cells, but also on normal B cells. It has been reported that patients treated with anti-CD20 antibodies had an insufficient response to two-dose mRNA SARS-CoV-2 vaccination. To investigate the efficacy of a third dose in these patients, we investigated serum IgG antibody titers for the S1 protein after a third vaccination in 22 patients treated with the anti-CD20 antibody who failed two-dose vaccination. Results showed that overall, 50% of patients seroconverted. Although no patient who received the third dose within 1 year of the last anti-CD20 antibody administration showed an increase in S1 antibody titer, 69% of patients who received the third dose more than 1 year after the last anti-CD20 antibody administration seroconverted. Our data show that a third dose of vaccination is effective in improving the seroconversion rate in patients treated with the anti-CD20 antibody who failed standard two-dose vaccination.

## 1. Introduction

Patients with hematological malignancies, especially those receiving anti-CD20 antibody therapy, have increased morbidity and mortality from coronavirus disease 2019 (COVID-19) infection [1,2,3]. Furthermore, because the anti-CD20 antibodies rituximab and obinutuzumab react with CD20 expressed not only on malignant B cells but also on normal B cells, they impair the efficacy of SARS-CoV-2 mRNA vaccination in triggering the humoral immune response [4,5]. In real-world settings, it has been reported that patients treated with anti-CD20 antibodies have an insufficient response after two-dose vaccination compared with an age-matched healthy cohort, and a third dose (booster) vaccination in these patients is accordingly expected to improve immunogenicity [6,7,8]. However, data on third-dose vaccination remain insufficient.

In our previous study, we investigated S1 antibody titers 2 weeks after the second dose of mRNA SARS-CoV-2 vaccination, BNT162b2, in patients with B cell malignancy who had been treated with the anti-CD20 antibody before vaccination [5]. Results showed that many patients (29 of 39) failed to achieve seroconversion [5]. In the present study, we investigated the efficacy of a third dose of vaccination in these non-seroconverting patients. Additionally, to investigate surrogate markers of antibody production ability, we investigated the relationship between the percentage of peripheral blood B cells (CD19-positive cells) or serum IgG level and S1 antibody titer.

## 2. Materials and Methods

### 2.1. Study Design

We previously investigated the immunogenicity of two-dose vaccination (BNT162b2) in patients with B-cell malignancies who had been treated with the anti-CD20 antibodies rituximab and obinutuzumab [5]. In this present study, we enrolled 22 patients in our previous study in whom the second dose failed to produce seroconversion (optimal optical density (O.D.) cut-off values of anti-S1 IgG antibody and anti-nucleocapsid IgG antibody for seroconversion were determined to be 0.26 and 0.7, respectively [9]) at Kobe University Hospital between April 2022 and June 2022. All participants were vaccinated with a third dose of mRNA SARS-CoV-2 vaccination (BNT162b2 or mRNA-1273). Peripheral blood samples were collected 14 days (+/−7 days) after the third dose of vaccination. Exclusion criteria included documented COVID-19 infection (positive PCR test result). Vaccine-related adverse events were evaluated using Common Terminology Criteria for Adverse Events 5.0, except for fever, which we defined as Grade 1, 37.5–37.9 °C; Grade 2, 38.0–38.9 °C; Grade 3, 39.0–39.9 °C; and Grade 4, >40.0 °C in the axilla.

The study protocol was approved by the Kobe University Hospital Ethics Committee (No. B2056714, 1481) and was conducted in accordance with the Declaration of Helsinki. All patients provided written informed consent to participate.

### 2.2. Sample Collection and Measurement of Antibody Titers against S1 Protein

Serum samples were obtained by centrifuging blood samples for 10 min at 1000× *g* at room temperature, and were immediately transferred to a freezer kept at −80 °C.

Antibody titers against S1 and nucleocapsid proteins were measured by the QuaResearch COVID-19 Human IgM IgG ELISA Kit (spike protein S1) (Cellspect, Inc., RCOEL961-S1, Iwate, Japan) and QuaResearch COVID-19 Human IgM IgG ELISA Kit (nucleocapsid protein) (Cellspect, Inc., RCOEL961-N, Iwate, Japan), respectively. These kits detected antibody titers based on the indirect ELISA method, and came with different immobilized antigenic proteins. The plate of the ELISA kit (spike protein S1) was immobilized with a recombinant spike protein (S1, 251-660AA) of SARS-CoV-2 expressed in Escherichia coli. The plate of the ELISA kit (nucleocapsid protein) was immobilized with a recombinant nucleocapsid protein (full length) of SARS-CoV-2 expressed in Escherichia coli. Serum samples were diluted 1:200 in 1% BSA/PBST for RCOEL961-S1 and 1:1000 in 1% BSA/PBST for RCOEL961-N. The plates were read at 450 nm with an SH-1200 plate reader (Corona Electric Co., Ltd., Hitachinaka, Japan) in accordance with the manufacturer’s measurement protocol.

## 3. Results

### 3.1. Patient Characteristics

We enrolled 22 patients treated with the anti-CD20 antibody who failed standard two-dose vaccination. All patients (median age 74 years, range 57–86) received a third dose of vaccination (BNT162b2, n = 18 and mRNA-1273, n = 4, respectively) 6–50 (median 18.5) months after the final dose of anti-CD20 antibody (rituximab, n = 21 and obinutuzumab, n = 1) (Table 1). Diagnoses included diffuse large B-cell lymphoma (n = 13), follicular lymphoma (n = 6), lymphoplasmacytic lymphoma/Waldenström macroglobulinemia (n = 2), and mantle cell lymphoma (n = 1) (Table 1). No patient had received any chemotherapy since the last anti-CD20 antibody dose. No patient had comorbidities for which immunosuppressive drugs were indicated. Serious adverse events (>Grade 3) following the third dose of vaccination were not observed.

### 3.2. Serological Outcomes

**Figure 1:**
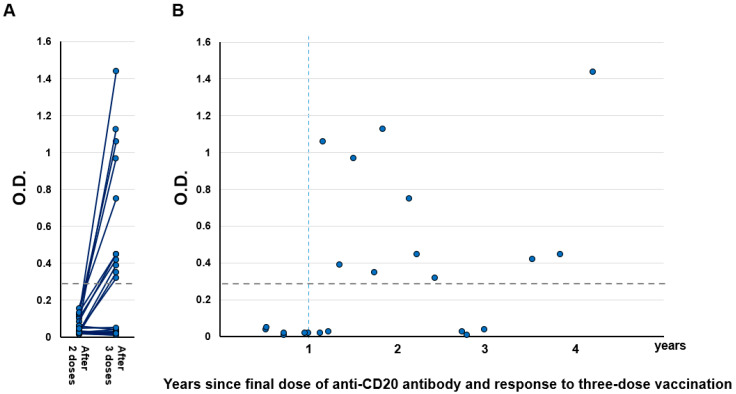
(**A**) Humoral quantitative anti-spike 1 (S1) antibody response 14 days (±7 days) after the second and third vaccination in B-cell lymphoma patients treated with anti-CD20 antibody (n  =  22). The horizontal dotted line indicates the threshold (0.26) for seroconversion. (**B**) Relationship between S1 titer and time from the final dose of anti-CD20 antibody to the third dose of vaccination. The horizontal dotted line indicates the threshold (0.26) for seroconversion. O.D.: Optical density.

S1 antibody titers were measured approximately 14 days after the third dose of vaccination (median 13 days, range 7–17) with a COVID-19 Human IgM IgG ELISA Kit (spike protein S1) (Cellspect, Inc., Morioka, Japan). Results showed that overall, 50% (11/22) of patients seroconverted (Figure 1A). Importantly, no patient who received the third dose within 1 year of the last anti-CD20 antibody administration showed an increase in S1 antibody titer (0/6, Figure 1B). In contrast, 69% patients (11/16) who received the third dose more than 1 year after the last anti-CD20 antibody administration seroconverted.

We also evaluated the anti-SARS-CoV-2 nucleocapsid protein IgG antibody in the same serum samples of all participants to check for previous COVID-19 infection. We confirmed that all participants did not achieve seroconversion of anti-nucleocapsid IgG (Supplemental Figure S1).

**Figure 2:**
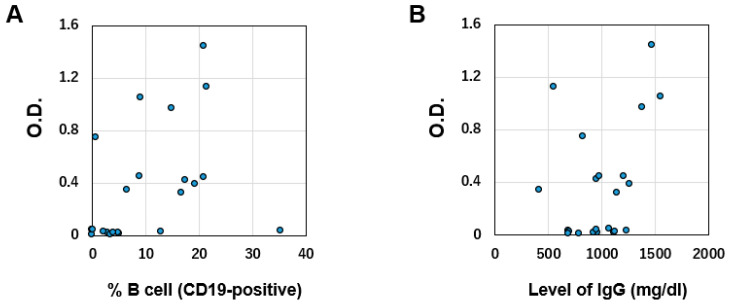
(**A**) Correlation between S1 antibody titer and percentage of CD19-positive B cells (normal range 6–23%). (**B**) Correlation between S1 antibody titer and total IgG level (normal range 870–1700 mg/dL). O.D.: Optical density.

We investigated surrogate markers of antibody production ability by vaccination. We found no relationship between the percentage of peripheral blood B cells (CD19-positive cells) or serum IgG level and S1 antibody titer (r2 = 0.10 and 0.14, respectively; Figure 2A,B).

## 4. Discussion

In this study, to test the efficacy of a third dose of vaccination in patients treated with the anti-CD20 antibody, we enrolled only patients who failed to seroconvert after a second dose of vaccination. Although there was concern that the intense immunosuppression caused by anti-CD20 antibodies could persist over the long term, most patients (69%) who received the third dose more than 1 year after the last anti-CD20 antibody administration seroconverted. This result shows that mRNA vaccination is indicated even in patients who have received B-cell depletion therapy. Nevertheless, some issues concerning the booster shot remain. First, seroconversion following the third dose was not seen in patients who had received their final dose of anti-CD20 antibody within one year previously. Second, about 30% of patients who had received their final dose of anti-CD20 antibody more than one year previously did not achieve seroconversion. Furthermore, the seroconversion rate did not appear to increase with increasing years after the final dose of anti-CD20 therapy. We previously reported that an increase in antibody titers following two-dose vaccination remained limited for a long time (more than 3 years) after the final dose of anti-CD20 antibody [5]. These results are consistent with the idea that long-term immunosuppression is maintained.

In these patients, an alternative protection method might be necessary. A study group reported that heterologous boosters increased immune response [10], and another group speculated that a second booster (fourth dose) gives immunocompromised patients extra protection from COVID-19 [11]. However, sufficient data are not yet available. At the present time, we recommend that these patients continue to wear a face mask and wash their hands to prevent COVID-19 even after vaccination.

Monitoring recovery of the ability to produce antibodies following vaccination requires consideration. Some groups have reported that the percentage of peripheral blood B cells predicts humoral immune response upon SARS-CoV-2 vaccination among patients treated with rituximab [12,13]. However, our data revealed no relationship between S1 antibody titers and % of B cells. Clarifying this issue will require a large-scale study; at present, monitoring this ability after vaccination might require the measurement of SARS-CoV-2 antibodies themselves.

This study has some limitations. First, we did not perform a neutralization assay. Although generally there was a significant and positive correlation between S1 antibody titers and neutralizing activity [14,15], analysis of neutralizing responses is important to confirm neutralizing responses in seroconverted patients. Second, because we did not enroll enough age-matched healthy volunteers, we did not perform a statistical evaluation between healthy volunteers and cancer patients. Finally, our sample size was small. Confirmation of these tendencies requires further investigation with a larger number of participants.

## 5. Conclusions

In conclusion, our data show that the third dose of vaccination is effective in improving the seroconversion rate in patients treated with the anti-CD20 antibody who failed standard two-dose vaccination. In terms of monitoring recovery of the ability to produce antibodies by vaccination, further studies are required.

## Figures and Tables

**Table 1:** Patient characteristics.

Total	n = 22
**Median age** **(range, years)**	74 (57–86)
**Sex**	
Female	8
Male	14
**Diagnosis**	
DLBCL	13
FL	6
LPL/WM	2
MCL	1
**Anti-CD20 antibody**	
Rituximab-based	21
Obinutuzumab-based	1
**Median course** **(range, courses)**	6 (3–20)

DLBCL: diffuse large B-cell lymphoma, FL: follicular lymphoma, LPL/WM: lymphoplasmacytic lymphoma/Waldenström macroglobulinemia, MCL: mantle cell lymphoma.

## Data Availability

The data presented in this study are available on request from the corresponding author.

## Supplementary Materials

The following supporting information can be downloaded at: https://www.mdpi.com/article/10.3390/vaccines10060965/s1, Figure S1: Humoral quantitative anti-SARS-CoV-2 nucleocapsid protein IgG antibody response in the same serum samples of all participants (B-cell lymphoma patients treated with anti-CD20 antibody, n = 22). The horizontal dotted line indicates the threshold (0.7) for seroconversion. O.D.: Optical density.
