# Supplementary figures and images for: Promising Efficacy of a Third Dose of mRNA SARS-CoV-2 Vaccination in Patients Treated with Anti-CD20 Antibody Who Failed 2-Dose Vaccination
Source: Vaccines (Basel). 2022 Jun 17;10(6):965. doi: 10.3390/vaccines10060965 (PMC9229139; doi:10.3390/vaccines10060965)

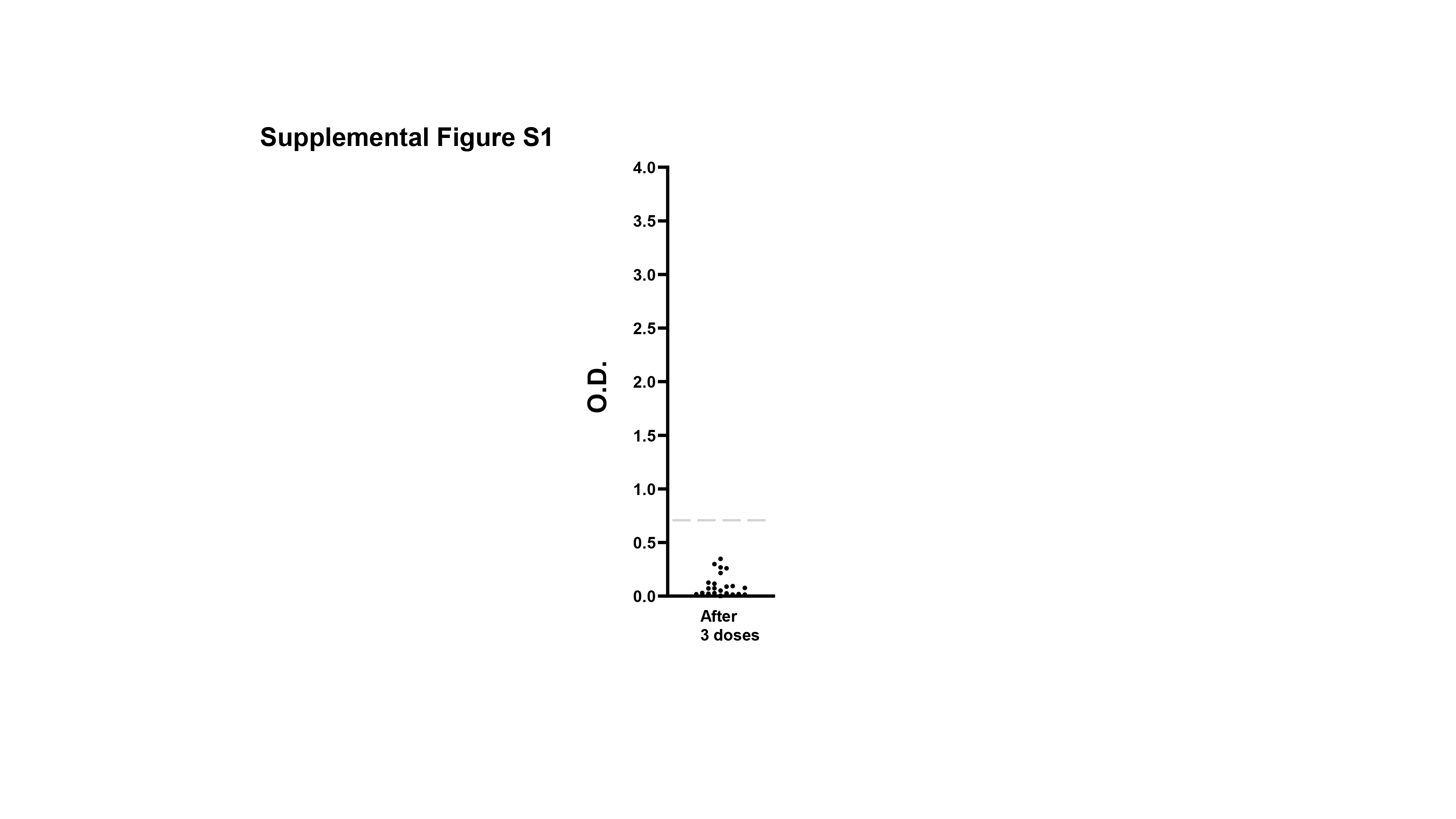

Supplement: Supplementary file 1 [file vaccines-10-00965-s001.zip › S1.tif]
